# Adaptation of Translational Machinery in Malaria Parasites to Accommodate Translation of Poly-Adenosine Stretches Throughout Its Life Cycle

**DOI:** 10.3389/fmicb.2019.02823

**Published:** 2019-12-06

**Authors:** Jessey Erath, Sergej Djuranovic, Slavica Pavlovic Djuranovic

**Affiliations:** Department of Cell Biology and Physiology, Washington University School of Medicine, St. Louis, MO, United States

**Keywords:** plasmodium, ribosomes, mRNA surveillance, AT rich genome, mRNA translation

## Abstract

Malaria is caused by unicellular apicomplexan parasites of the genus *Plasmodium*, which includes the major human parasite *Plasmodium falciparum*. The complex cycle of the malaria parasite in both mosquito and human hosts has been studied extensively. There is tight control of gene expression in each developmental stage, and at every level of gene synthesis: from RNA transcription, to its subsequent translation, and finally post-translational modifications of the resulting protein. Whole-genome sequencing of *P. falciparum* has laid the foundation for significant biological advances by revealing surprising genomic information. The *P. falciparum* genome is extremely AT-rich (∼80%), with a substantial portion of genes encoding intragenic polyadenosine (polyA) tracks being expressed throughout the entire parasite life cycle. In most eukaryotes, intragenic polyA runs act as negative regulators of gene expression. Recent studies have shown that translation of mRNAs containing 12 or more consecutive adenosines results in ribosomal stalling and frameshifting; activating mRNA surveillance mechanisms. In contrast, *P. falciparum* translational machinery can efficiently and accurately translate polyA tracks without activating mRNA surveillance pathways. This unique feature of *P. falciparum* raises interesting questions: (1) How is *P. falciparum* able to efficiently and correctly translate polyA track transcripts, and (2) What are the specifics of the translational machinery and mRNA surveillance mechanisms that separate *P. falciparum* from other organisms? In this review, we analyze possible evolutionary shifts in *P. falciparum* protein synthesis machinery that allow efficient translation of an AU rich-transcriptome. We focus on physiological and structural differences of *P. falciparum* stage specific ribosomes, ribosome-associated proteins, and changes in mRNA surveillance mechanisms throughout the complete parasite life cycle, with an emphasis on the mosquito and liver stages.

## Introduction

**FIGURE 1:**
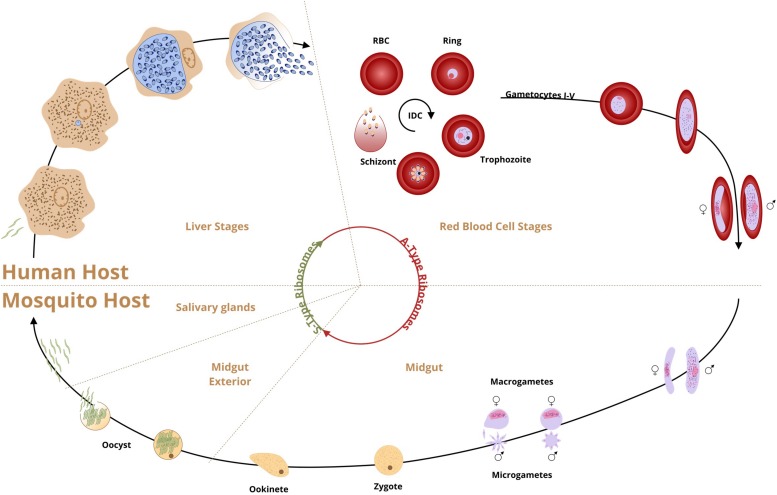
Schematic presentation of *P. falciparum* life cycle and stage-specific ribosomal RNAs (A and S type). Human infection by *P. falciparum* begins when an infected female anopheline mosquito inoculates sporozoites into the bloodstream during feeding. The sporozoites invade liver cells and transform into trophozoites. In 6–8 days one mature schizont will release thousands of liver-stage merozoites into the bloodstream (exoerythrocytic schizogony, the first proliferative stage). The second asexual proliferative stage blood stage (erythrocytic schizogony) starts when the liver-stage merozoites invade the erythrocytes About 14–16 erythrocytic merozoites are generated in a 48-h cycle for re-infection and it is the point when the symptoms start. The merozoites may differentiate into single gametocytes, the initial stage of the sexual reproduction (gametogenesis) or continue the asexual cycle. Mosquito infection begins when the gametocytes are taken by mosquito with the blood meal. The male microgametocyte exflagellates into individual microgametes and fertilizes the female macrogamete. The zygote transforms into a motile ookinete, penetrates the mosquito midgut and develops into an oocyst. After 9–14 days, thousands of sporozoites are differentiated in the mature oocyst (sporogony), the only multiplicative stage in the mosquito. Mature sporozoites invade salivary glands and with the next blood meal the cycle continues.

*Plasmodium* spp. has been in existence long before humans were on Earth, with an estimated origin of malaria-causing parasites appearing around 165 million years ago. Consequently, mosquitos and malaria had millions of years to co-evolve before either ever interacted with humans (Winegard, 2019). The infection of humans occurred evolutionarily recently, and probably with multiple *Plasmodium* parasite species. *P. falciparum* and *P. vivax* established themselves as a major malaria causing species. *P. falciparum* a most virulent agent in human malaria began speciation around 50,000 years ago followed by the population bottleneck around 5000 years ago but higher level of genetic diversity suggests that *P. vivax* is older (Loy et al., 2018; Otto et al., 2018). *P. malariae*, *P. ovale*, and rare cases of *P. knowlesi* were also reported in human hosts. From the mid-19th century onward, malaria reached its global limits and exacted immensely high numbers in sickness and death. While increased malaria prevention and control treatments have reduced the health burden of malaria, there are still 219 million cases of infection per year resulting in a 435,000 deaths (World Health Organization [WHO], 2018). The complex cycle of the malaria parasite in both mosquito and human hosts has been studied extensively (Figure 1). In each of these life cycle stages, gene expression is tightly controlled (Le Roch et al., 2004; Shock et al., 2007; Hughes et al., 2010; Sorber et al., 2011; Bunnik and Le Roch, 2013; Caro et al., 2014; Vembar et al., 2016a; Lu et al., 2017).

It took years of laborious efforts to sequence *P. falciparum* genome (Kooij et al., 2006). Sequences of single or multiple chromosomes as well as complete genome were reported over the course of 4 years (Gardner et al., 1998, 2002a,b; Bowman et al., 1999; Hall et al., 2002; Hyman et al., 2002). The high AT-content of the genome made gap closure in sequences extremely difficult. However, long-read, single molecule, real-time sequencing allowed for complete telomere-to-telomere *de novo* assembly of the *P. falciparum* genome thereby overcoming the problems associated with next generation sequencing of AT-rich genomes (Vembar et al., 2016b). The consequence of AT-richness is the presence of extended tracts of As, Ts, and TAs in introns and intergenic regions (Glöckner, 2000; Szafranski et al., 2005) as well as unusually high number of genes containing coding polyadenosine (polyA) repeats compared to the other species (Habich, 2016; Djuranovic et al., 2018). Repetitions of 12 or more adenosine nucleotides in gene coding sequences, so-called polyA tracks, were recently found to act as negative gene regulation motifs at the level of mRNA translation in all tested organisms (Arthur et al., 2015; Koutmou et al., 2015). Consequently, polyA tracks have been evolutionarily preserved in a select set of genes, but are generally selected against in overall gene coding sequences (Arthur et al., 2015).

Recent analysis of 250 eukaryotic genomes found a median of 2% of transcripts with polyA tracks (Habich, 2016). However, *Plasmodium* species represent an exception to this rule. The percentage of polyA carrying transcripts in the genome exceeds 60% for most *Plasmodium* spp., including *P. falciparum* (64%) (Djuranovic et al., 2018). The pervasive ribosomal stalling and frameshifting found on polyA tracks in other eukaryotes (Arthur et al., 2015; Koutmou et al., 2015; Tournu et al., 2019) would make it almost impossible for the majority of *Plasmodium* proteins to be efficiently and correctly synthesized. However, global studies of *Plasmodium* protein composition (Florens et al., 2002; Silvestrini et al., 2010) and protein synthesis (Le Roch et al., 2004; Bunnik et al., 2013; Caro et al., 2014) do not show any reduction in either the protein or mRNA abundances of polyA track genes. This suggests that both ribosomal stalling and frameshifting in *Plasmodium* are resolved by adaptations in protein synthesis and mRNA quality control systems. In this review, we will discuss how the extreme AT-rich genome of malaria-causing parasite promoted special features in *P. falciparum* ribosomes to enable translation of polyA tracks throughout the complete life cycle. Additionally, genomic changes and parasitic environment have also influenced variation in mRNA surveillance mechanism within the organism resulting in divergence from other Eukaryotes.

## Evolution of At-Richness in *P. falciparum*

Extremes in genomic base composition toward GC- or AT-richness exist in all domains of life (Sueoka, 1962; Wernegreen and Funk, 2004; Zilversmit et al., 2010; Wu et al., 2012). The extent of these extremes in nucleotide composition is limited by the necessity of all 20 amino acids and the subsequent requirement of all four nucleotides to encode them. As such, long homopolymeric amino acid repeats appear to be a characteristic of genomes with either bias (Glöckner, 2000; Albà et al., 2007; Muralidharan et al., 2011). Harboring either extreme AT- or GC-richness affects genomic structure, stability, transcriptome, and codon bias of organisms (Wu et al., 2012). As seen in Table 1, the *P. falciparum* mean AT-richness of around 80% appears to be one of the highest in all Eukaryotes (Pollack et al., 1982; Musto et al., 1999; Gardner et al., 2002a; Videvall, 2018). Surprisingly, the higher AT-content of the *P. falciparum* genome cannot be fully explained by increased AT-richness in intergenic regions, but rather by contributions of AT-richness in both coding 76.22% (Table 1) and non-coding genome 90% (Gardner et al., 2002a). Overall, gene organization patterns in *P. falciparum* are not influenced by the AT-bias (Glöckner, 2000; Szafranski et al., 2005; Djuranovic et al., 2018). However, what distinguishes *Plasmodium* species from other AT-rich organisms is distribution of consecutive adenosine nucleotides resulting in unusually high percentage of polyA track genes (Table 2). The genomes of *P. falciparum* and related *Plasmodium* species have apparently evolved independently to reach extreme AT-bias (Table 2). Interestingly, while the two groups of *Plasmodium* species can be separated based on their AT-genomic content (median of 75% versus a median of 55% AT-richness), both groups accommodate a considerable amount of polyA tracks within the coding regions (Djuranovic et al., 2018).

**TABLE 1:** Comparison of AT-richness and polyA track gene ratios over selected *Eukaryotic* species.

**Organism**	**CDS % AT richness**	**PolyA track genes**
*Plasmodium falciparum*	76.22%	63.54%
*Plasmodium reichenowi*	75.93%	62.93%
*Dictyostelium discoideum*	72.57%	20.96%
*Tetrahymena thermophila*	72.50%	28.19%
*Saccharomyces cerevisiae*	60.39%	5.51%
*Plasmodium knowlesi*	59.77%	41.42%
*Caenorhabditis elegans*	57.94%	1.93%
*Plasmodium vivax*	53.51%	38.85%
*Drosophila melanogaster*	50.66%	1.10%
*Pan troglodytes*	50.60%	2.17%
*Homo sapiens*	49.98%	1.40%
*Trypanosoma brucei*	49.19%	2.89%
*Trypanosoma cruzi*	46.83%	2.74%
*Toxoplasma gondii*	42.88%	1.01%
*Leishmania donovani*	37.63%	0.19%
*Leishmania major*	37.52%	0.05%
*Leishmania infantum jpcm5*	37.50%	0.12%
*Acanthamoeba castellanii str neff*	37.06%	0.11%
*Emiliania huxleyi*	31.38%	0.10%
*Aureococcus anophagefferens*	29.37%	0.33%

*The coding region AT-richness from a relevant selection of organisms with high, moderate, and low AT-content was compiled from Habich (2016) and Videvall (2018) and sorted in descending order.*

**TABLE 2:** Comparison of AT-Richness and polyA track gene ratios over selected *Plasmodium* species.

**Organism**	**CDS % AT richness**	**PolyA track genes**
*Plasmodium gallinaceum*	78.81%	71.77%
*Plasmodium relictum*	78.43%	79.18%
*Plasmodium berghei*	76.26%	68.26%
*Plasmodium yoelii 17x*	77.03%	64.96%
*Plasmodium falciparum*	76.22%	63.54%
*Plasmodium chabaudi*	74.46%	63.37%
*Plasmodium reichenowi*	75.93%	62.93%
*Plasmodium vinckei petteri*	74.91%	62.58%
*Plasmodium gaboni*	77.56%	61.76%
*Plasmodium falciparum camp malaysia*	76.71%	61.02%
*Plasmodium falciparum nf54*	76.55%	60.60%
*Plasmodium falciparum fch 4*	76.67%	60.57%
*Plasmodium falciparum ugt5 1*	76.49%	60.45%
*Plasmodium falciparum santa lucia*	76.75%	60.42%
*Plasmodium falciparum palo alto uganda*	76.56%	60.33%
*Plasmodium falciparum nf135 5 c10*	76.56%	59.98%
*Plasmodium falciparum malips096 e11*	76.50%	59.81%
*Plasmodium falciparum 7g8*	76.65%	59.72%
*Plasmodium yoelii yoelii*	75.20%	51.06%
*Plasmodium knowlesi*	59.77%	41.42%
*Plasmodium knowlesi strain h*	59.76%	41.42%
*Plasmodium vivax*	53.51%	38.85%
*Plasmodium cynomolgi strain b*	57.91%	33.83%
*Plasmodium inui san antonio 1*	56.37%	31.12%

**Plasmodium* spp. coding region AT-content and the ratio of polyA affected transcripts was collected (Habich, 2016; Videvall, 2018). The data are organized in the table to demonstrate a separation of two groups with high and low coding region AT-content and subsequently the number of polyA track containing transcripts. The separation notably occurs along the line of geographic region with the high AT-content organisms being predominantly found in Africa and the low AT-content group in Asia, Southeast Asia, and Latin America. However, the low AT-content group still exceeds that of most organisms.*

Perhaps just as interesting as the consequences of genomic base composition biases are the factors driving it. Previous studies in *P. falciparum* were unable to conclude the primary role of homopolymeric amino acid repeats in the parasite proteome (Muralidharan et al., 2011; Muralidharan and Goldberg, 2013). Nutrient availability to intracellular parasites – as well as endosymbionts – appears to be a major factor in driving AT-richness, particularly nitrogen availability (Seward and Kelly, 2016; Dietel et al., 2019). *De novo* synthesis of nucleotides comes at great metabolic expense, especially regarding G+C nucleotides (Dietel et al., 2019). A+T nucleotides are less metabolically costly to create and tend to be more abundant. Consequently, A+T nucleotides are easier to scavenge, even in intracellular environments where nutrients may not be readily available. In the case of *P. falciparum*, where *de novo* synthesis of purines does not occur, the parasites must rely upon purine scavenging and salvage pathways (Ting et al., 2005; El Bissati et al., 2006; Quashie et al., 2008). Conversely, pyrimidine *de novo* synthesis occurs using glutamine and aspartic acid precursors. This appears to be the main source for these nucleotides, with the folate pathway being required for thymidine production (Sherman, 1979; Cassera et al., 2011; Hamilton et al., 2017). However, unlike other intracellular organisms referenced above, the intracellular environment for *P. falciparum* is not necessarily nutrient poor, but perhaps nutrient selective; particularly prior to parasite augmentation of the host cell. While *P. falciparum* has multiple means by which amino acids are obtained, much of its initial amino acid supply is from proteolysis of human host red blood cell hemoglobin (Leiriao et al., 2004; Liu et al., 2006; Babbitt et al., 2012).

This brings us to a second major contributor of AT-richness in intracellular organisms: oxidative stress. Reactive nitrogen (RNS) and reactive oxygen species (ROS) generate oxidative stress resulting in 8-oxoguanine production via guanine oxidation. If left unrepaired in DNA, 8-oxoG is able to pair with adenosine; ultimately causing a G:C to T:A conversion. Compounding the process, hemoglobin degradation produces free heme and H_2_O_2_, which generates further oxidative stress for the parasite (Becker et al., 2004). Additionally, NO and other RNS species may be important factors in the soluble heme-hemozoin equilibration (Ostera et al., 2011). Interestingly, another erythrocytic parasite from *Apicomplexa* phylum, *Babesia microti*, does not degrade hemoglobin and has a considerably less AT-rich genome (61.02%) and polyA tracks (2.17% of genes with polyA tracks) compared to *P. falciparum* (Cornillot et al., 2012; Djuranovic et al., 2018). Although *Plasmodium* spp. does supply some of its own antioxidants to cope with oxidative assault, the higher than expected G:C to T:A conversion in the organism suggests a lack of full compensation by the biochemical and/or DNA repair safeguards (Hamilton et al., 2017). While 8-oxoG could potentially result in AT-richness imprinted in the DNA sequence, it causes more problems when found in RNA (Simms and Zaher, 2016). The oxidative lesion and incorporation of 8-oxoG in mRNAs reduces the rate of peptide-bond formation by more than three orders of magnitude (Simms et al., 2014). The effect of 8-oxoG nucleotides in mRNAs is independent of its position within the codon, results in stalling of the translational machinery, and finally activation of No-Go decay mRNA surveillance mechanisms (Simms et al., 2014). As such, the presence of oxidative stress may have driven both an increase in genomic AT-richness and changes in mRNA surveillance mechanisms of *P. falciparum*; which are discussed further below.

AT-richness itself appears to provide a feedback loop in the parasite with its increased indel rates, which are thought to be due to DNA replication slippage on AT repeats. These AT tracks provide amplicon breakpoints for copy number variant (CNV) alteration via non-allelic homologous repair-like mechanism that can be advantageous in altering resistance gene CNV numbers (Guler et al., 2013; Hamilton et al., 2017; Huckaby et al., 2019). Altogether, metabolic and biochemical factors continuously drive the parasite genome toward AT richness, which, in turn, drives indels that potentiate genomic plasticity providing an overall platform for relatively rapid adaptive evolution in the parasite. Unarguably, these factors necessitate increased fidelity in DNA replication and RNA transcription. While the exact details specific to *Plasmodium* spp. evolutionary adaptation toward an AT-rich genome, unique codon biases, and polyA encoded lysine stretches remains to be explored, the role ribosomes play as influential factors in this process is certain.

## The rRNA and Specialized Ribosomes of *Plasmodium*

The most abundant genes in cells and genomes from bacteria to eukaryotes are those encoding ribosomal RNA. Ribosomal RNA genes in eukaryotic cells form clusters with a highly repetitive structure. *S. cerevisiae*, a single cell organism, has 150 rDNA repeats in one cluster on chromosome XII, while human cells contain five clusters of approximately 70 rDNA repeats on five different chromosomes (Sakai et al., 1995). The organization of rDNA genes in clusters is conserved among most of the eukaryotic organisms (Kobayashi, 2014). Transcription of these clusters is highly coordinated to meet the huge demand for ribosomes, which occupy ∼50% of the total protein mass in a cell (Warner, 1999). *Plasmodium* genomes, however, have only 4–8 single copy rDNA units that are encoded on different chromosomes (Gunderson et al., 1987; Waters et al., 1989; Li et al., 1997). Such a small number of rDNA copies throughout the genome is seen elsewhere only in bacteria. *E. coli* has seven ribosomal RNA genes spread over its circular genome and well positioned in the regions near an origin of replication. This arrangement in *E. coli* enables maximum ribosomal RNA transcription while preventing possible collisions between replication forks and transcription machinery (Ellwood and Nomura, 1982). Thus, while most of the other organisms have optimized ribosome production, how the malaria-causing parasite produces its significant ribosome numbers is still unknown. It might be possible that massive DNA replication that occurs throughout its lifecycle (during shizogeny) in both hosts may accommodate the rRNA production requirements.

Besides this unusual rDNA arrangement, malaria parasites are pioneers in the new era of specialized ribosomes (Walliker et al., 1987; McCutchan et al., 1988; Waters et al., 1989; Velichutina et al., 1998; Xue and Barna, 2012). *Plasmodium* spp. has structurally distinct, stage-specific ribosomes and are the most well-known case of rRNA heterogeneity (McCutchan et al., 1988). The difference in sequence and expression profile during the life cycle classified them into A-type (asexual stage specific) and S-type (sporozoite specific) in the majority of *Plasmodium* species, including *P. falciparum*; with *P. vivax* having a third O-type rRNA (Li et al., 1997). The A-type is present in the liver and blood stage and S-type is sporozoite specific rRNA type that emerges during the mosquito stage and ends during the parasite development in hepatocytes (Zhu et al., 1990). Here, we will focus on the process by which the ribosome types switch and whether ribosomes with distinct rRNA play a selective role in the mRNAs they translate.

*Plasmodium* spp. have adapted to translation in two different hosts. This requires translation optimization at two distinct temperatures, one of which can be highly variable depending on the mosquito environment. Even though one would think that changes in temperature and hosts would be the reason for development of different rRNAs, the presence of A-type during the early mosquito stage and S-type during early liver stage does not support that idea (Fang and McCutchan, 2002). The rRNA sets are not expressed in an exclusive and binary (on/off) fashion, but more as a dynamic, heterogeneous population whereby one subtype, A or S, is the more dominant rRNA type in a particular lifecycle stage. While the idea of a thermoregulatory nature of the rRNA units has been explored earlier in *P. berghei*, rodent malaria, it has not been followed since (Fang and McCutchan, 2002). *P. berghei*, contains four distinct copies off the rRNA (A, B, C, D) and they are divided into A-type (A and B) and S-type (C and D). A single copy of the S-type gene, C or D was sufficient for life cycle completion, which only affected the parasite fitness. The group was unable to disrupt both S-type genes simultaneously; nor could they disrupt either of A-type genes (van Spaendonk et al., 2001). Interestingly, authors noticed growth retardation in oocyst development, which was more pronounced in D-unit disruption rather than in C-unit (van Spaendonk et al., 2001). Such difference could be explained by difference in ribosomal levels stemming from different transcriptional levels of C- and D-units or functional diversity of C- and D-unit containing ribosomes (Xue and Barna, 2012; Mills and Green, 2017). The disruption of specific S-type rRNA is also associated with oocyst development defects in the second rodent parasite *P. yoelii* (Qi et al., 2015). Finally, van Spaendonk et al. (2001) note a lack in differences between core catalytic components (e.g., GTPase center) of the ribosome large subunit in *P. berghei* that were previously described in *P. falciparum* (Velichutina et al., 1998). These results among species of *Plasmodium* potentiate the question of some aspect of ribosomal specialization (Vembar et al., 2016a).

**FIGURE 2:**
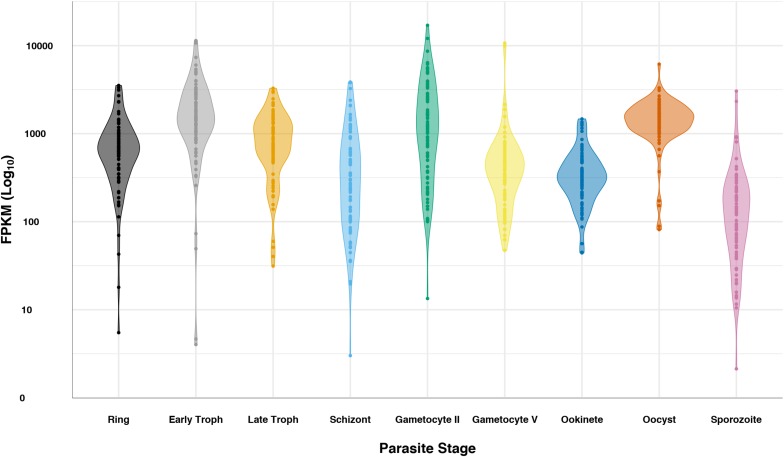
*Plasmodium falciparum* ribosomal protein expression over life cycle. RNAseq data (López-Barragán et al., 2011; Zanghì et al., 2018) for the 82 cytosolic ribosomal proteins was queried from PlasmoDB (Aurrecoechea et al., 2009) for ring, early trophozoite, late trophozoite, schizont, gametocyte II, gametocyte V, ookinete, oocyst, and sporozoite stages. Data is displayed in log scale as a violin plot, showing the general trend of ribosomal protein transcripts. Data for each ribosomal protein is represented as a dot in the plot.

Previous bacterial work has shown changes in rRNA operon expression in response to stress, resulting in phenotypic changes (Kurylo et al., 2018). The change in *Plasmodium* spp. rRNA population dynamics in response to environmental stress from host transfer is reminiscent of the bacterial changes in rRNA operon expression. However, whether changes in ratios of *Plasmodium* spp. rRNA types drive phenotypic changes is still unknown. Ostensibly, the ribosomes share the same repertoire of ribosomal proteins. RNAseq data shows that while ribosomal protein gene transcription as a whole is fairly persistent throughout the complete life cycle of *P. falciparum*, oscillations in their overall expression pattern match that of stages with increased protein synthesis (Figure 2). This does not exclude the highly sought-after notion that a specific set of ribosomes may be optimized for specific mRNA substrates or cell populations that may also exist in *Plasmodium* spp. A recent study in zebrafish showed that embryos have different subtypes of 5.8S, 18S, and 28S rRNAs, creating similar ribosome diversity seen in *Plasmodium* cells (Locati et al., 2017). *In silico* data have shown that the expanded regions of 18S subunit expressed in zebrafish embryos may preferentially bind maternal transcripts when compared to somatic subtypes (Locati et al., 2017). Similarly, a shift in the expression of 16S rRNA ribosome variants created populations of *E. coli* cells that accommodated functional differences in tetracycline binding (Kurylo et al., 2018). As was mentioned before, the rRNA heterogeneity that was mostly known in *Plasmodium* parasites (Gunderson et al., 1987; Waters et al., 1989; Zhu et al., 1990; Rogers et al., 1996; Xue and Barna, 2012) is now recognized in other organisms (Locati et al., 2017; Kurylo et al., 2018). However, the role of different *Plasmodium* rRNAs as a response to different environmental conditions is still not defined.

## *Plasmodium* Ribosomes, Polya and Poly-Lysine Sequences

**FIGURE 3:**
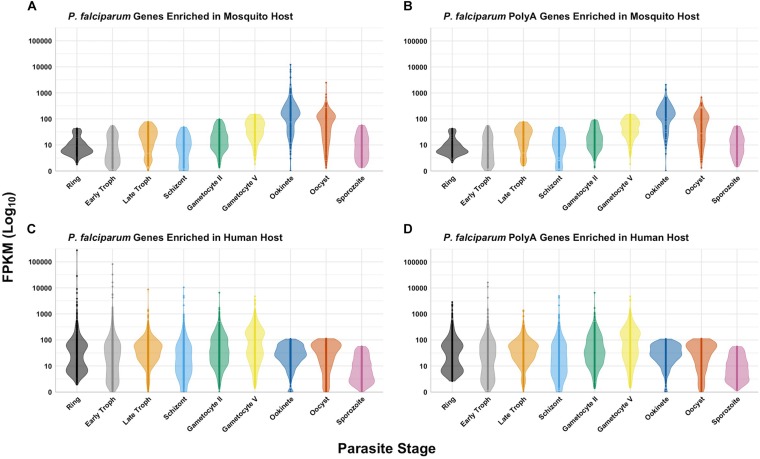
Expression of *P. falciparum* genes in different hosts. The data was queried from PlasmoDB (Aurrecoechea et al., 2009; López-Barragán et al., 2011; Zanghì et al., 2018) for protein-coding genes with expression data greater than or equal to the 80th percentile for ring, early trophozoite, late trophozoite, schizont, gametocyte II, gametocyte V, ookinete, oocyst, and sporozoite stages. *P. falciparum* genes enriched in the mosquito host **(A)**, human host **(C)**, and polyA genes for each (**B** and **D**, respectively) are as previously defined. RNAseq data for all stages in both hosts for these gene sets are displayed in log scale as a violin plot with all data points for comparison.

**FIGURE 4:**
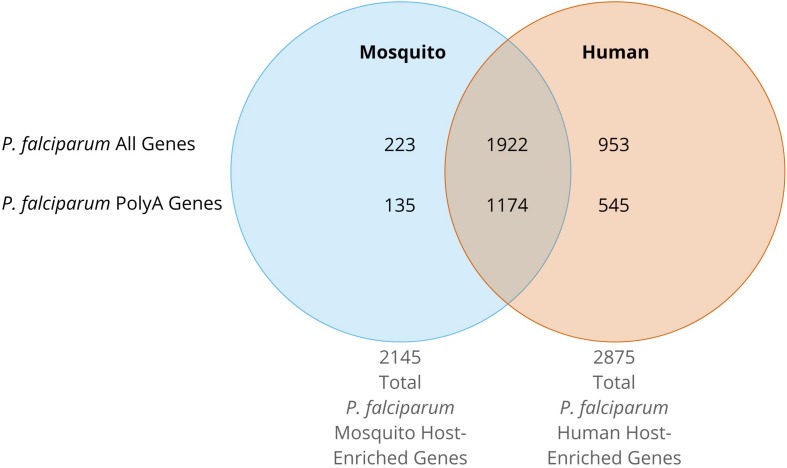
Venn Diagram of *P. falciparum* gene expression from RNAseq data. The expression data greater than or equal to the 80th percentile for ring, early trophozoite, late trophozoite, schizont, gametocyte II, gametocyte V, ookinete, oocyst, and sporozoite stages was queried (López-Barragán et al., 2011; Zanghì et al., 2018). Mosquito stage total genes were defined as those with expression data greater than or equal to the 80th percentile during gametocyte V, ookinete, oocyst, or sporozoite stages. Mosquito host-specific genes are defined as above, but solely in mosquito host stages. Total parasite genes expressed in the human host were defined as those with expression data greater than or equal to the 80th percentile during sporozoite, ring, early trophozoite, late trophozoite, schizont, gametocyte II, or gametocyte V stages. Enriched parasite genes expressed in the human host are defined as above, but solely in human host stages. PolyA genes are those defined as having one or more runs of twelve or more consecutive adenosines in the coding region of the gene.

Regardless of the host, all *Plasmodium* spp. rRNA types must contend with the translation of unusually high AU-content and long-coding polyA stretches in mRNAs. RNA-seq data (Le Roch et al., 2004; Shock et al., 2007; Bunnik et al., 2013; Guler et al., 2013; Caro et al., 2014) indicates that the mRNA levels of genes containing polyA stretches follows the same trend as the general gene expression for all stages in both hosts (Figures 3A–D). We can conclude that both types of ribosomes expressed in both hosts have features allowing efficient translation of transcripts containing long, coding polyA tracks. This indicates that *P. falciparum* ribosomes have higher fidelity during translation of polyA sequences and are able to accommodate long polybasic peptides coming through their protein-exit channel. Previous ribosome mutagenesis studies in *S. cerevisiae* suggested functional differences in the GTPase centers of *P. falciparum* A- and S-type ribosomes (Velichutina et al., 1998). Despite the differences in yeast viability and growth rates, chimeric yeast ribosomes with either *Plasmodium’s* A- or S-type GTPase centers exhibited increased translational accuracy (Velichutina et al., 1998). Even though there are stage-specific ribosomes, there is a group of genes that is present in human and mosquito that contain polyA tracks (Figure 4). More recently it was also shown that the *P. falciparum* ribosomes have been altered to accommodate the poly-lysine patches that are prolific throughout the proteome (Djuranovic et al., 2018). To allow these low-complexity, homopolymeric and polybasic amino acid repeats, the parasite ribosome exit channel has been altered by increasing the channel size at key bottle necks, as well as a reduction in the hydrophobicity patches typically seen in bacterial, yeast, or human ribosomes (Djuranovic et al., 2018).

Ribosome profiling and biochemical assays suggest an increased or modified fidelity such that parasite ribosomes do not stall or frameshift on polyA tracks (Djuranovic et al., 2018). The mechanism of this altered fidelity may result from not only modification of the ribosomal RNA sequence, but also via changes to key protein components of ribosomes. Two *P. falciparum* ribosome cryoEM structures suggest a reduced or lost interaction of the receptor for activated C kinase 1 (RACK1) to *Plasmodium* ribosomes (Wong et al., 2014; Sun et al., 2015). RACK1 has been established as an integral ribosomal scaffold protein (Sengupta et al., 2004). Beside other non-ribosome associated functions, RACK1 was found to be important for cap-dependent translation initiation, IRES-mediated translation, and site-specific translation (Majzoub et al., 2014). RACK1 also contributes to the translation arrest that is induced by translation of polyA sequences (Dimitrova et al., 2009; Kuroha et al., 2010), CGA-CGA codons in yeast (Wolf and Grayhack, 2015), or runs of consecutive basic amino-acid (Kuroha et al., 2010). Stalls on polyA tracks can be resolved in mammalian cells by deletion of RACK1 and ZNF598, thus enabling read-through of stall-inducing sequences (Garzia et al., 2017; Juszkiewicz and Hegde, 2017; Sundaramoorthy et al., 2017). *S. cerevisiae* ribosomes lacking the RACK1 homolog Asc1 are able to translate through the CGA-CGA stalling sequences and increase normally attenuated protein output (Wolf and Grayhack, 2015). The increase in amount of synthesized protein from CGA-CGA sequences is a consequence of overall reduced elongation rates of yeast ribosomes that lack Asc1 (Tesina et al., 2019). Slower elongation rates may also influence cellular responses to ribosome pausing. The position of RACK1/Asc1 near the mRNA exit channel on the ribosome could be important in sensing ribosome collisions that lead to activation of ribosome rescue and mRNA surveillance pathways (Kim et al., 2014; Simms and Zaher, 2016; Tesina et al., 2019). The fact that *Plasmodium* ribosomes lack interaction with PfRACK1 could be beneficial for translation of polyA tracks into poly-lysine runs. However, based on previous conclusions concerning the role of RACK1/Asc1 in correct reading frame maintenance during translation of stalling sequences, the majority of polyA coding sequences in malaria parasites would be predicted to have multiple frameshifted protein products.

Previous studies (Lu and Deutsch, 2008; Kuroha et al., 2010; Brandman et al., 2012) proposed that stalling during the translation of polyA tracks is due to synthesis of the poly-lysine rich nascent peptide. Electrostatic interactions of the polybasic peptide and the peptide exit tunnel in the ribosome would elicit ribosomal stalling (Lu and Deutsch, 2008). Recent studies revealed that an mRNA-mediated mechanism is directly contributing to stalling (Arthur et al., 2015; Koutmou et al., 2015; Tesina et al., 2019). Consecutive adenosines are engaged by the ribosome decoding center nucleotides, are stabilized on both sides by rRNA base stacking interactions (Tesina et al., 2019), and adopt a helical conformation typical for single stranded polyA stretches (Tang et al., 2019). PolyA tracks are highly efficient at causing ribosome stalling, and the inhibitory conformation of polyA mRNA bases can further contribute to a polyA-mediated stalling mechanism. This conclusion is in line with the previous observations that consecutive AAG codons are less efficient at causing stalling than AAA codons (Arthur et al., 2015; Koutmou et al., 2015), despite coding for the same amino acid. Altogether, the charge and conformation of the poly-lysine nascent chain in conjunction with the stacked polyA mRNA nucleotides in the decoding center of the ribosome contribute to the overall stalling mechanism (Tesina et al., 2019). *P. falciparum* ribosomes are again the exception to this rule. The nucleotides that make stacking interactions with polyA repeats are conserved in *P. falciparum* ribosomes. However, both endogenous transcripts and reporter sequences with long runs of polyA tracks are efficiently translated by *Plasmodium* (Lacsina et al., 2011; Bunnik et al., 2013; Djuranovic et al., 2018). Thus, in order to adapt to polyA track translation for production of the polybasic and homopolymeric lysine repeats, the malaria-causing parasite has altered the sequence of its rRNA, its ribosome structure, its ribosomal proteins, and its mRNA translation quality control pathways.

## mRNA Surveillance Pathways in Au-Rich Transcriptome of *P. falciparum*

The core elements for mRNA translation are highly conserved in *Plasmodium* spp. (Vembar et al., 2016a*)*. The unique features involving protein synthesis in *Plasmodium*, such as different types of ribosomes in different life cycle stages, were noticed even before genome sequencing of the malaria parasite (Gunderson et al., 1987; Zhu et al., 1990; Rogers et al., 1996; van Spaendonk et al., 2001). However, the presence of an unusual number of mRNA binding proteins and the absence of some elements of mRNA surveillance mechanism were noticed upon completion of the *P. falciparum* genome sequence (Gunderson et al., 1987; Waters et al., 1989; Rogers et al., 1996; van Spaendonk et al., 2001; Le Roch et al., 2004; Bunnik and Le Roch, 2013; Cui et al., 2015; Reddy et al., 2015; Lu et al., 2017). A recent review elaborated on the translational regulation in blood-stages of malaria parasites (Vembar et al., 2016a). They focused on cytoplasmic mRNA translation and the fate of mRNAs: decoding of the mRNA messages by the 80S ribosomes, degradation of mRNAs by exo- or endo-nucleases (mRNA decay), and sequestration of mRNAs by protection from mRNA decay or by inhibition of translation. We focus here on the mechanism of activation of mRNA surveillance pathways by aberrant mRNAs in the context of unusual AU-richness and abundance of polyA tracks in *Plasmodium* transcriptome.

Eukaryotic cells have developed mechanisms to protect themselves from the production of the possible toxic proteins due to aberrant mRNA translation events. There are three mRNA quality control systems for translational errors in eukaryotes: Non-sense mediated decay (NMD), No-Go decay (NGD), and Non-Stop decay (NSD). NMD targets transcripts harboring “premature” termination codons (PTC) and nascent polypeptide chains synthesized from such transcripts for efficient degradation (Shoemaker and Green, 2012). Components of NMD pathway distinguishes PTCs from authentic stop codons in the coding sequence. PTCs are usually the product of point-non-sense mutations, ribosomal frameshifting on slippery sequences, aberrant splicing events, or in some cases, the consequence of targeted gene regulation through alternative splicing (Sorber et al., 2011; Yeoh et al., 2019). In higher eukaryotes, PTCs are generally recognized by their proximity to so-called exon-junction complexes (EJCs), which are deposited near exon junctions during pre-mRNA splicing in the nucleus (Shoemaker and Green, 2012).

No-Go decay is an “umbrella term” for the mRNA surveillance pathway that deals with either damaged or difficult to translate mRNA sequences that cause the ribosome to stall during the elongation cycle of translation. Besides the previously mentioned mRNA base damages (8-oxoG) (Simms et al., 2014; Simms and Zaher, 2016), mRNA translation can be stalled by lack of aminoacylated-tRNAs, strong mRNA secondary structure (i.e., stem-loops or long GC-rich regions), or stable interaction of the nascent polypeptide chain with the translating ribosome. Even though Non-Stop Decay (NSD) was discovered earlier than NGD (Doma and Parker, 2006; Izawa et al., 2012; Tsuboi et al., 2012; Saito et al., 2013; Martin et al., 2014; Guydosh and Green, 2017), it became apparent that in mammals and higher eukaryotes, the NSD and NGD pathways share the same effector protein complexes (Saito et al., 2013). The NSD targeted mRNAs that originate from premature 3′ adenylation or cryptic polyadenylation signals found in coding sequences indeed represent a similar group of transcripts that would be targeted by NGD pathway (Saito et al., 2013; Kashima et al., 2014; Martin et al., 2014). Ribosomes that translate mRNAs without stop codons would eventually stall while translating long polyA tails into poly-lysine repeats, or because they would simply run out of message. Recognition of these types of transcripts, as well as the aforementioned NGD targets, trigger components of NGD/NSD pathways resulting in targeted mRNA cleavage and degradation.

The majority of mRNA surveillance pathway genes have been annotated in the *P. falciparum* genome (Table 3; Hughes et al., 2010). However, there are no mechanistic studies to confirm the activity of these pathways. Most of our knowledge on *Plasmodium*’s mRNA surveillance pathways comes from bioinformatic searches using homologous sequences from other eukaryotes. An indirect proof of the existence of NMD in *Plasmodium* is through the studies of alternative splicing of pre-mRNA (Sorber et al., 2011; Yeoh et al., 2019). Regulated alternative splicing events generating transcripts that do not lead to apparent protein synthesis usually carry PTCs, and thus are committed to NMD. Alternative splicing in *P. falciparum* has been reported for several genes like delta-aminolevulinic acid dehydratase (ALAD), stromal processing peptidase (SPP), and chloroquine resistance transporter (*Pf*CRT); among the others. Additionally, studies on the essentiality of *Plasmodium* genes that use the CRISPR/Cas9 technique (Ghorbal et al., 2014) or transposon techniques (Zhang et al., 2018) rely partially on silencing targeted genes through the activation of NMD. In this case, activation of NMD is the consequence of either mutations that are generated during CRISPR/Cas9 DNA cleavage, transposon insertion in the coding sequence, or due to aberrant splicing events caused by transposons landing in introns of targeted genes. As noted above, more than 60% of the *P. falciparum* transcripts harbor polyA track motifs that are seen as mRNA “slippery” sequences during translation (Habich, 2016; Djuranovic et al., 2018). Translation of runs of poly-adenosine nucleotides results in ribosomal frameshifting in most tested organisms causing activation of NMD pathways (Arthur et al., 2015; Koutmou et al., 2015). However, ribosome profiling (Lacsina et al., 2011; Bunnik et al., 2013) and reporter assays (Djuranovic et al., 2018) indicate that *P. falciparum* ribosomes maintain fidelity during translation of rather long polyA stretches (more than 36As in a row). Therefore, while there is indirect evidence that the NMD pathway exists in *Plasmodium*, it seems that this pathway is not upregulated during *Plasmodium* ribosomes’ interactions with its polyA runs and AU-rich coding sequences. The most probable reason for this is the above mentioned changes in ribosome structure and fidelity.

**TABLE 3:** Comparison of translation quality control factors in *P. falciparum* and its mosquito and human hosts.

**Pathway**	***H. sapiens***	***A. gambiae***	***P. falciparum***
NMD	eRF1, eRF3, UPF1, UPF2, UPF3A/UPF3B, eIF4AIII, MLN51, Y14/MAGOH, BTZ, SMG1, SMG5, SMG6, SMG7, PP2, Musashi, PABP1	eRF1 (AGAP010310), eRF3 (AGAP009310), UPF1 (AGAP001133), UPF2 (AGAP000337), UPF3 (AGAP006649), eIF4AIII (AGAP003089), Y14 (AGAP006365)/MAGOH (AGAP010755), SMG1(AGAP000368), SMG5 (AGAP008181), SMG6 (AGAP000894), PP2A (AGAP004096), Musashi (AGAP001930)	UPF1 (PF3D7_1005500), UPF2 (PF3D7_0925800), UPF3B (PF3D7_1327700), eIF4AIII (PF3D7_0422700) PF3D7_1327700), PP2A(PF3D7_0925400 – KEGG, PF3D7_1319700 – Hs homology, or PF3D7_0927700 – name), Musashi (PF3D7_0916700), PABP1 (PF3D7_1224300), eRF1 (PF3D7_0212300), eRF3 (PF3D7_1123400)
NGD/NSD	Pelota/HBS1L, RACK1, ZNF598, N4BP2 (Cue2)	Pelota (AGAP008269), HBS1L (AGAP002603), RACK1 (AGAP010173), ZNF598 homolog (AGAP007725), N4BP2 homolog (AGAP002516)	Pelota (PF3D7_0722100), RACK1 (PF3D7_0826700), ZNF598 (PF3D7_1450400)
RQC	CNOT4, ABCE1, TRIP4, ASCC2, ASCC3, NEMF, Listerin, UBE2D1, XRN1	CNOT4, ABCE1 (AGAP002182), ASCC2 homolog (AGAP000428), ASCC3 (AGAP001234), NEMF homolog (AGAP002680), Ltn1 (AGAP007143), UBE2D1 homolog (AGAP000145), XRN1	ABCE1 (PF3D7_1368200), NEMF homolog (PF3D7_1202600), Listerin homolog (PF3D7_0615600), CNOT4 (PF3D7_1235300), ASCC3 homolog (PF3D7_1439100), UBE2D1 homolog (PF3D7_1203900) XRN1 (PF3D7_0909400)

*Factors associated with NMD, NGD/NSD, and RQC pathways from the literature and KEGG pathway database in human cells were collected (Kanehisa et al., 2019). Homologs in *A. gambiae*, one of the most common and effected vectors of P. *falciparum* (Cohuet et al., 2006; Annan et al., 2007; Giraldo-Calderon et al., 2015), were collected using KEGG pathways and performing protein-BLAST searching using VectorBase. Confirmation to FlyBase was also used to confirm vague annotations. *P. falciparum* factors were similarly documented again using KEGG pathways and PlasmoDB protein-BLAST analysis (Aurrecoechea et al., 2009; Kanehisa et al., 2019; Thurmond et al., 2019). Homologous gene database IDs are listed for reference. Notably, NGD/NSD factors Hbs1L and Cue2 endonuclease are missing in *P. falciparum* genome.*

Genomic sequencing has also revealed several critical components of surveillance pathways that are missing. According to NCBI, KEGG, and plasmoDB databases, *P. falciparum* and the majority of other *Plasmodium* spp. lack the NGD and NSD decay pathways components Hbs1 (Doma and Parker, 2006) and Cue2-RNA endonuclease (D’Orazio et al., 2019). With the exception of *S. cerevisiae*, the Hbs1/Pelo protein complex rescues stalled ribosomes on mRNAs. It was postulated that stalling events cause ribosome collisions (Simms et al., 2017), generating unique disome units consisting of the stalled ribosome and the following colliding ribosome (Beckman and Inada). The disome, as a minimal ribosome collision unit, is recognized by Ribosome-associated Quality Control (RQC) and NGD pathways (Ito-Harashima et al., 2007; Izawa et al., 2012; Tsuboi et al., 2012; Guydosh and Green, 2017; Juszkiewicz and Hegde, 2017). Activation of RQC and NGD leads to cleavage of stalled mRNA by Cue2, and possibly other unknown endonucleases, which ultimately leads to ribosome rescue by the activity of the Pelo/Hbs1 complex (Ito-Harashima et al., 2007; Tsuboi et al., 2012; Kashima et al., 2014; Matsuda et al., 2014; Sugiyama et al., 2019). In most of the above mentioned RQC and NGD studies, a typical substrate for ribosomal stalling is a long polyA run, ranging from 36 to 60 adenosines, coding for a peptide with 12–20 consecutive lysine residues. However, long polyA stretches in *P. falciparum* cells are efficiently translated into poly-lysine repeats (Lacsina et al., 2011; Bunnik et al., 2013). Of note, the longest endogenous polyA runs in different *P. falciparum* species range from 88 to 111 nucleotides and code for *Plasmodium* specific and hypothetical proteins (Habich, 2016), which is longer than the length of the normal 3′ polyA tail in either *S. cerevisiae* or human cells (Brown and Sachs, 1998; Chang et al., 2014; Subtelny et al., 2014). As such, many endogenous *Plasmodium* transcripts would be NSD targets in other eukaryotic organisms. It is also a question as to what the signal for NSD pathway is in *Plasmodium* as recent study on 3′ mRNA polyadenylation in apicomplexans did not find any differences in *P. falciparum* polyadenylation complex, polyA binding proteins, or polyA tails when compared to other species (Stevens et al., 2018; Kanehisa et al., 2019). Because *Plasmodium* lacks the components to rescue stalled ribosomes, and because *Plasmodium* ribosomes efficiently translate long polyA runs, the function and mechanism of the NGD/NSD pathway in *P. falciparum* remains a mystery.

## Conclusion

While it may seem reasonable that *P. falciparum* adapted its ribosomes for higher fidelity on polyA runs and in parallel lost the ability to activate the RQC/NGD/NSD pathways, such a scenario is far from obvious. The absence of mRNA surveillance pathway components or deletion of RQC factors leads to both protein aggregation and proteotoxic stress in yeast cells (Choe et al., 2016; Yonashiro et al., 2016; Jamar et al., 2018). Protein aggregation is observed in *P. falciparum* in the absence of heat shock protein 110 (Muralidharan et al., 2012) but not due to the absence of mRNA surveillance or RQC pathways or as a consequence of increase in both number or length of polyA tracks (Djuranovic et al., 2018). This conflicting result, along with the surprising lack of interaction between the ribosomal scaffold protein RACK1/Asc1 and *Plasmodium* ribosomes (Wong et al., 2014; Sun et al., 2015), argue that the mRNA surveillance pathways in *P. falciparum* are inherently different from those in other eukaryotes. The diversity of rRNAs, *Plasmodium’*s ribosome structure, and the activity of yet unknown ribosome associated factors promote the possibility of “specialized ribosomes” in *Plasmodium* that allow for polyA tracks translation into functional proteins. Each of the aforementioned changes in parasites translational machinery and mRNA quality control pathways come at the cost of self-fitness that would normally be detrimental for survival of *Plasmodium* parasites in both humans and mosquitos. And yet the parasite has persisted in both of these hosts for hundreds of millions of years. Parasitologists and epidemiologists have wondered “How?” for decades; now as translational biologists, we add our voices to the same question.

## Author Contributions

All authors contributed equally to writing of this review.

## Conflict of Interest

The authors declare that the research was conducted in the absence of any commercial or financial relationships that could be construed as a potential conflict of interest.
